# Right femoral vein and right dorsal artery thrombosis in childhood acute myeloid leukemia: A case report

**DOI:** 10.1097/MD.0000000000035121

**Published:** 2023-10-13

**Authors:** Jiaqi Ni, Min Chen, Yali Su, Qianqian Gao, Lingjun Liu, Xiaoxi Lu

**Affiliations:** a Department of Pharmacy/Evidence-Based Pharmacy Center, West China Second University Hospital, Sichuan University, Chengdu, China; b Key Laboratory of Birth Defects & Related Diseases of Women and Children, Sichuan University, Ministry of Education, Chengdu, China; c West China School of Pharmacy, Sichuan University, Chengdu, China; d Department of Pediatric Hematology and Oncology, West China Second University Hospital, Sichuan University, Chengdu, China; e Department of Ultrasound, West China Second University Hospital, Sichuan University, Chengdu, China; f Department of Radiology, West China Second University Hospital, Sichuan University, Chengdu, China.

**Keywords:** acute myeloid leukemia, arterial thrombosis, case report, children, deep vein thrombosis

## Abstract

**Background:**

It is rare for newly diagnosed (de novo) or newly treated acute myeloid leukemia (AML) complicated with thrombotic complications, especially combined arterial and venous thrombosis.

**Methods:**

We reported a 13-year-old boy diagnosed with AML and leukocytosis, who developed right femoral vein and right dorsal artery thrombosis during chemotherapy. After treatment with low molecular weight heparin, diosmin, and alprostadil, symptoms were relieved. Unfortunately, the child suffered from coagulopathy afterward, which was unexpectedly caused by vitamin K deficiency.

**Results:**

After supplementation with vitamin K and prothrombin complex concentrate, coagulation function recovered.

**Conclusion:**

For childhood AML patients with high thrombotic risks, close monitoring during anticoagulant treatment was necessary. Concomitantly, we should be alert to past medication history and combined medication use, especially those that may lead to vitamin K deficiency, secondary bleeding, and coagulation disorders. Rational use of antibiotics, anticoagulants, and antitumor drugs must be guaranteed.

## 1. Introduction

Acute myeloid leukemia (AML) accounts for around 15% to 20% of childhood acute leukemia, which is a malignant disease of the hematopoietic system. AML often presents with fever, anemia, hemorrhage, lymphadenopathy, and hepatosplenomegaly.^[1]^ In the past 20 to 30 years, the survival rate of childhood AML has improved significantly, with an overall survival rate of 60% to 70%, and an event-free survival rate of over 50%.^[2–7]^ Due to the prothrombotic nature of cancer and its associated coagulopathies as well as the use of chemotherapeutic agents, the use of central venous catheters, surgery, and radiotherapy, children with cancer tend to have increased risks of thromboembolism (TE). The prevalence of cancer-associated thrombosis varies from up to 16% of symptomatic to 40% of asymptomatic TE.^[8]^ A population-based cohort study showed that the absolute incidence of venous thromboembolism (VTE) in children with cancer was 1.52 per 1000 person-years, while those without cancer had only 0.06 per 1000 person-years, with a risk ratio of 28.3.^[8]^ The incidence of arterial thrombosis in children with cancer is far lower than that of venous thrombosis.^[9]^ In addition, the incidence of TE was higher in hematologic tumors compared with solid tumors.^[10]^ We reported the clinical characteristics, pathogenesis, treatment, and prognosis of a child with AML complicated with arterial and venous thrombosis.

## 2. Case presentation

### 2.1. Case presenting

A 13-year-old boy presented to the emergency room with dizziness for 6 days. Physical examination revealed: T: 36.4°C, P: 88 times/min, RR: 17 times/min, BP: 106/61 mm Hg. The superficial lymph nodes, liver, and spleen were not palpable or swollen. The dorsum of the right foot was swollen with tenderness. No relevant family or past medical history was identified.

### 2.2. Laboratory examination

On admission, the whole blood test was remarkable for white blood cell (WBC) count of 174.8 × 10^9^/L, hemoglobin 120 g/L, platelet count 174 × 10^9^/L, blast percentage: 95%. Bone marrow smear indicated AML-M1. Immunohistochemistry showed: AML with HLA-DR, CD4, CD7, CD13, CD33, CD34, CD38, CD58, CD117, CD123 expression. AML common fusion gene test demonstrated: CLAM-AF10 positive. Gene mutation analysis revealed WT1 somatic mutation (c.1400G>C), FLT3-ITD somatic mutation (frequency ≥ 0.5), and TP53 germline mutation (c.886C>T). The FISH test showed negative results regarding MLL and D7Z1/D7S486 mutations.

Urea: 2.6 mmol/L, creatinine: 48 µmol/L, and uric acid: 197 µmol/L. Disseminated intravascular coagulation (DIC) test revealed: prothrombin time (PT): 14.7 seconds, activated partial thrombin time (APTT): 27.8 seconds, fibrinogen (Fib): 469 mg/dL, thrombin time (TT): 15.6 seconds, D-dimer: 1.34 mg/L FEU, fibrinogen degradation product (FDP): 4.17 µg/mL, antithrombin III (AT-III): 88%.

### 2.3. Treatment and outcome

**Figure 1.**
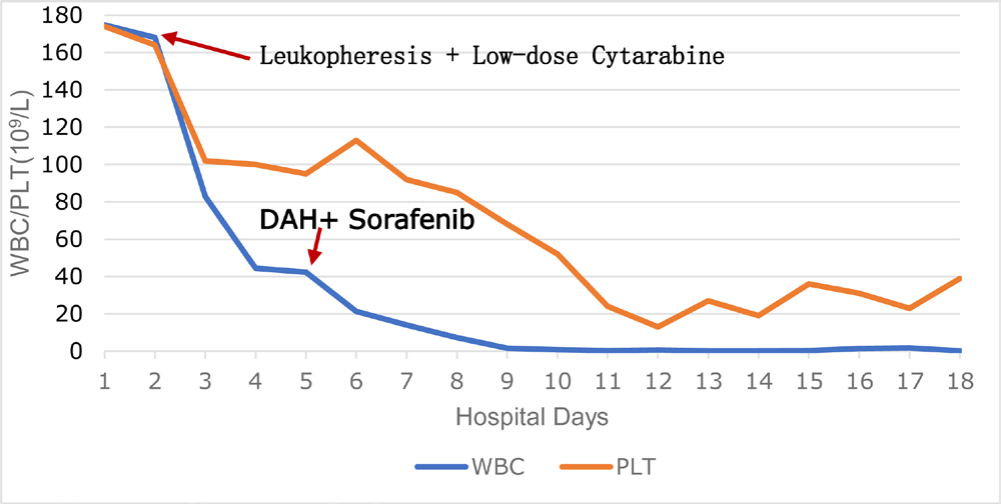
White blood cell and platelet changes during chemotherapy. DAH = daunorubicin, cytarabine, and homoharringtonine, PLT = platelet, WBC = white blood cell.

The child was diagnosed with AML combined with leukocytosis. The femoral vein of the right lower limb was intubated, then leukapheresis with a combination of low-dose cytarabine was initiated to lighten the tumor burden. WBC dropped remarkably after treatment (Fig. 1).

**Figure 2.**
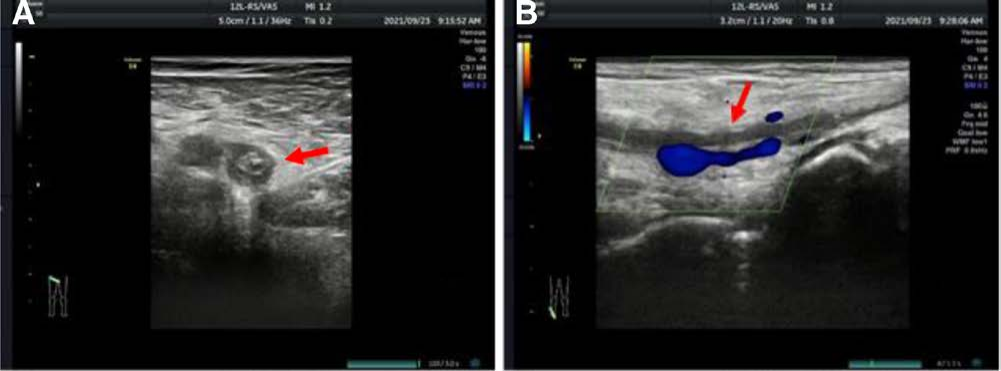
Two-dimensional ultrasonography showed a noncompressible right femoral vein containing hyperechogenicity debris, suggesting right femoral vein thrombosis (A). The right dorsal artery exhibited no blood flow with the color Doppler, indicating right dorsal artery thrombosis (B).

According to the CCLG-AML-2019 treatment protocol,^[11]^ induction chemotherapy including daunorubicin, homoharringtonine, cytarabine, and sorafenib was administered to the patient. During the first course of induction chemotherapy, the child developed intolerable right foot pain with numbness, especially in the right toe. Physical exam revealed an absence of palpable distal pulses on the right lower extremity. The right foot was swollen, and slightly cooler to touch than the left foot; discoloration of skin overlying the right toe was noticed. Two-dimensional ultrasonography showed a noncompressible right femoral vein containing hyperechogenicity debris, suggesting right femoral vein thrombosis. The right dorsal artery exhibited no blood flow with the color Doppler, indicating right dorsal artery thrombosis (Fig. 2). The patient was treated with low molecular weight heparin (LMWH) 6000IU subcutaneously once every 12 hours, alprostadil 10 µg intravenously once daily, diosmin 0.9 g orally 3 times daily, mucopolysaccharide polysulfate cream for external use, and local infrared radiation. After 10 days of treatment, the patient regained palpable pedal pulses with a resolution of his lower extremity symptoms. After consultation with the vascular surgery team, LMWH was suspended after 3 weeks of treatment.

**Figure 3.**
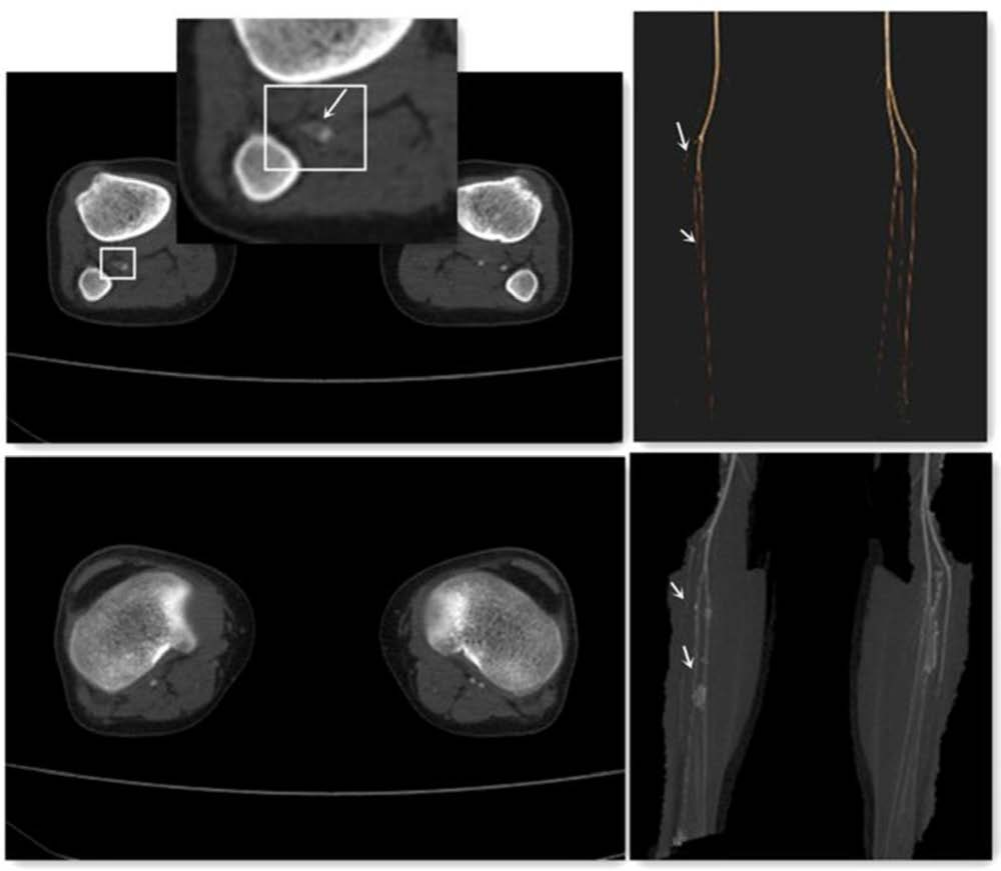
CTA showed that the lumen of the right popliteal artery branch and the beginning of the right anterior tibial artery with filling defects. The right dorsal artery is not visible. CTA = computed tomography angiography.

Unfortunately, 3 days after LMWH withdrawal, the child complained of acute onset of right big toe pain and numbness. A computed tomography angiography was obtained, which demonstrated occlusion of the right popliteal artery branch and the beginning of the right anterior tibial artery but also with thrombus occluding the right dorsal artery (Fig. 3). LMWH was continuously administered, and the DIC tests were performed regularly.

**Figure 4.**
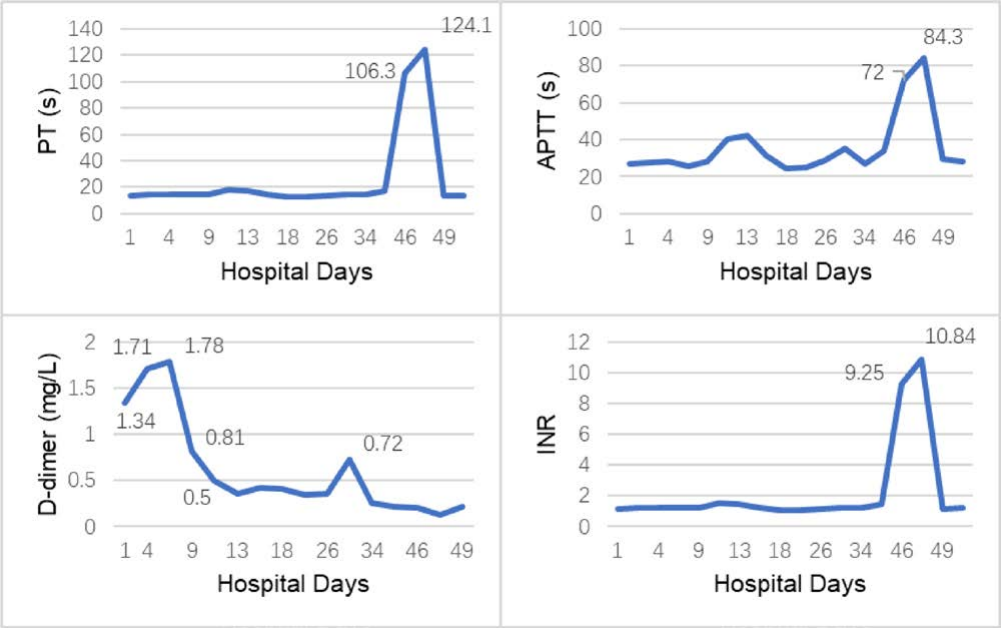
Lab tests of coagulation function during chemotherapy. APTT = activated partial thromboplastin time, INR = international normalized ratio, PT = prothrombin time.

After the first course of chemotherapy, bone marrow smears revealed the presence of 55% blasts, which indicated AML no remission. The second course of chemotherapy including idarubicin, homoharringtonine, cytarabine, and sorafenib was initiated. Within 2 weeks, however, the child developed febrile neutropenia, with WBC 0.1 × 10^9^/L, ANC 0.01 × 10^9^/L, C-reaction protein (CRP) 169.8 mg/L, and T 39.2°C. Cefoperazone and voriconazole were selected as the initial empirical antibiotic therapy based on the American Society of Clinical Oncology guideline.^[12]^ As the blood culture indicated gram-positive streptococcus mitis infection, vancomycin and linezolid were given successively. Unfortunately, a breakthrough occurred with chest pain, dry cough, and fever. Chest computed tomography confirmed invasive fungal disease, and then amphotericin B liposome was initiated. At the end of the second course of chemotherapy, the child still complained of right big toe pain and numbness/tingling. DIC test showed: PT 124.1 seconds, APTT 84.3 seconds, TT 15.3 seconds, DDI 0.13 mg/L FEU, FDP 1.28 µg/mL, INR 10.84 (Fig. 4). Considering the possibility of LMWH overload, the anti-Xa level was monitored with 0.1 IU/mL, a normal value that excluded LMWH overcorrection. Further tests were performed on coagulation factor levels, and a remarkable drop in Factor IX (F-IX) level (10.6%) was noticed. The child was on bed rest for a long time, with poor food intake, and long-term antibiotic use, so the lack of vitamin K, which further led to the F-IX synthesis defect was suspected. LMWH was suspended, and vitamin K combined with prothrombin complex concentrate was administered. After 2 days of treatment, the DIC test revealed normal coagulation function with PT 13.7 seconds, APTT 29.3 seconds, INR 1.14, TT 15 seconds, DDI 0.21 mg/L FEU, and FDP 1.65 µg/mL. After 3 weeks of treatment, no obvious thrombus was detected in the right lower extremity artery by ultrasound.

At the end of the second course of chemotherapy, a bone marrow smear revealed a blast percentage of 53%, which indicated no remission after 2 courses of induction chemotherapy. A combination of cladribine, cytarabine, granulocyte colony-stimulating factor, mitoxantrone, and sorafenib was administered. Eventually, a bone marrow smear after the third course of chemotherapy indicated complete remission, with MRD <0.01%. Afterward, the child received haploidentical hematopoietic stem cell transplantation and achieved continuous complete remission.

## 3. Discussion

The pathogenesis of leukemia complicated with thrombosis is multifactorial. First, leukemic cells produce procoagulant products such as tumor coagulant, tissue factor, and cytokine, thus inducing a coagulation cascade reaction, inducing the formation of thrombin and fibrin, and secondary DIC. Second, the direct damage of chemotherapy drugs to epidermal cells will weaken the antithrombotic effect. Chemotherapy drugs such as asparaginase were known to raise thrombosis risks.^[13,14]^ In addition, CVC-associated embolism is a common complication of childhood leukemia, which accounts for nearly half of VTE events.^[13–15]^ A retrospective study involving 652 children with acute lymphoblastic leukemia revealed that 57 children (8.7%) developed VTE, of which 74.1% occurred during induction chemotherapy, and only 5 cases occurred before asparaginase use. The vast majority of children had central venous catheters at the initial stage of treatment, and CVC-associated VTE occurred in 72.4% of children. VTE occurred most commonly in the subclavian vein, single venous thrombosis, jugular vein, fetal vein, upper limb, pulmonary embolism, and cardiac thrombosis.^[16]^ Another cohort study of 778 children with acute lymphoblastic leukemia showed that 59 children (7.6%) had VTE, of which 44.1% had cerebral venous sinus thrombosis; 59.3% (n = 35) of children had VTE during induction chemotherapy, and 40.7% (n = 24) had VTE during the medium-risk intensification period.^[17]^ Moreover, in patients with leukocytosis (WBC >100 × 10^9^/L), thrombosis risks rose since blood viscosity increased, blood flow speed slowed down, and peripheral blood leukocytes infiltrated in large amounts.^[18–22]^ Guo et al found that normal karyotypes with NPM1, and/or FLT3-ITD mutations were related to increased DIC risks in patients with nonacute promyelocytic leukemia, presenting with PT prolongation and D-dimer elevation.^[22]^ Additionally, previous VTE history, platelet counts >100 × 10^9^/L, and smoking are also risk factors for thrombosis in AML patients receiving chemotherapy.^[13]^ In this case, the child was diagnosed with AML-M1 with FLT3-ITD mutation, the WBC at admission was as high as 174.8 × 10^9^/L, platelet counts 174 × 10^9^/L, a catheter was placed in the femoral vein of the right lower limb, and received chemotherapy after admission. All of these factors raised the thrombosis risk for the patient.

Anticoagulation is complicated for children with cancer as this population is not only at risk of thrombosis but at risk of bleeding because of chemotherapy-related thrombocytopenia and coagulation disorders. Patients with symptomatic thrombosis should be given anticoagulation therapy without contraindications such as active bleeding. Thrombolytic therapy may be required in case of arterial thrombosis that endangers life or affects important organs.^[23]^ British Society for Hematology guideline recommends against routine thromboprophylaxis for children with cancer but can be considered for those with multiple risk factors. Anticoagulant treatment should be given for at least 3 months for symptomatic VTE. For those with active cancer or combined risk factors, a treatment period of over 3 months may be considered. In comparison with warfarin, LMWH is more suitable as an antithrombotic therapy for children with cancer. The peak concentration of the anti-Xa factor needs to be monitored regularly when on LMWH and maintained between 0.5 and 1.0 IU/mL. For children with high risks of bleeding, intravenous injection of unfractionated heparin with a shorter half-life is recommended.^[15]^

At the beginning of hospitalization, the child in this case was found thrombus around the common femoral vein catheter in the right lower limb, and occlusion of the right dorsal artery. After 10 days of treatment with LMWH combined with alprostadil and diosmin, the coagulation function turned normal, and the imaging revealed no thrombus. Unfortunately, while on continued anticoagulation treatment, the child presented with coagulopathy at a later stage, PT 124.1 seconds, APTT 84.3 seconds, and INR as high as 10.84. The child has no subcutaneous hemorrhage, hematemesis, hematochezia, or other bleeding manifestations, and it is difficult to interpret the cause of abnormal coagulation function. The normal anti-Xa level excluded the possibility of LMWH overdose. With a low F-IX level of 10.6%, the increase in PT, APTT, and INR was considered to be triggered by the lack of coagulation factors. Thus, LMWH was suspended, and vitamin K and prothrombin complex concentrate (coagulation factor II/VII/IX/X complex) were supplemented. The child has been on bed rest for a long time and experienced severe bone marrow suppression, sepsis blood flow infection, and malnutrition after 2 courses of chemotherapy. In addition, the child received intravenous antibacterial and antifungal drugs for a long time during 3 months of hospitalization, resulting in intestinal flora imbalance and further decreased synthesis of vitamin K-dependent factors, including factor II, VII, IX, and X, which caused coagulopathy. A cohort study involving 23,242 patients suggested that the use of cephalosporins, especially those with side chains containing N-methyl-thio-tetrazole (NMTT), such as cefoperazone, could lead to PT prolongation, coagulation dysfunction, thrombocytopenia, and even bleeding.^[24]^ Its mechanism is still unclear, which may be related to the consumption of vitamin K by NMTT group metabolism, the inhibition of intestinal flora resulting in intestinal flora disorder, the inhibition of vitamin K epoxide reductase, and the deficit of vitamin K synthesis. For ICU pediatric patients with inadequate vitamin K intake, a weekly supplementation of 0.3 mg/kg vitamin K was recommended by the British Society for Haematology guideline.^[25]^

## 4. Conclusion

Venous thrombosis, especially combined with arterial thrombosis, is rare in newly diagnosed or newly treated children with AML. Clinicians should be extremely vigilant, and take measures to reduce or eliminate thrombosis risks. Once thrombosis is confirmed, a multidisciplinary approach is recommended, including a prompt initiation of anticoagulation treatment. Concomitantly, coagulation function should be routinely monitored. The occurrence of coagulopathy is directly related to long-term bed rest, long-term use of antibiotics, malnutrition, and changes in intestinal flora.^[26–28]^ Therefore, possible contributing factors should be thoroughly investigated when coagulation disorder occurs. For example, vitamin K deficiency, which affects the synthesis of vitamin K-dependent anticoagulation factors in this case.

## Author contributions

All authors read and approved the final article.

**Participated in the coordination of the study:** Y.S., Q.G., L.L., and X.L.

**Collected and reviewed the medical records of the case:** J.N. and M.C.

**Conceptualized the study and conceived the study idea:** J.N., M.C., and X.L.

**Contributed to the data curation:** J.N. and M.C.

**Contributed to the funding acquisition:** J.N. and X.L.

**Contributed to the methodology:** J.N. and L.L.

**Wrote the original draft:** J.N., M.C., and X.L.

**Reviewed and edited the article:** J.N., M.C., Y.S., Q.G., L.L., and X.L.

**Contributed to the investigation:** M.C. and Q.G.

**Contributed to the resources:** Y.S., Q.G., L.L., and X.L.

**Contributed to the software:** Y.S., Q.G., and L.L.

**Contributed to the validation and visualization:** Y.S.

**Contributed to the formal analysis:** Q.G.

**Contributed to the project administration and supervision:** X.L.
